# Multimorbidity patterns and hospitalisation occurrence in adults and older adults aged 50 years or over

**DOI:** 10.1038/s41598-022-15723-4

**Published:** 2022-07-08

**Authors:** Luciana Pereira Rodrigues, João Ricardo Nickenig Vissoci, Diego Galdino França, Nayara Malheiros Caruzzo, Sandro Rogério Rodrigues Batista, Cesar de Oliveira, Bruno Pereira Nunes, Erika Aparecida Silveira

**Affiliations:** 1 Postgraduate Program in Health Sciences, Faculty of Medicine, Federal University of Goiás, Goiânia, 74605-050 Brazil; 2 Duke Global Health Institute, Duke University, Durham, NC USA; 3 State University of Maringá, Maringá, Brazil; 4 Faculty of Medicine, Federal University of Goiás, Goiânia, Brazil; 5 Department of Epidemiology and Public Health, University College London, London, UK; 6 Faculty of Nursing, Federal University of Pelotas, Pelotas, Brazil; 7 Department of Health, Government of Goiás, Goiânia, Brazil

**Keywords:** Diseases, Health care

## Abstract

Multimorbidity is highly prevalent in older adults and can lead to hospitalisation. We investigate the prevalence, associated factors, and multimorbidity pattern associated to hospitalisation, readmission, and length of stay in the population aged 50 years and older. We analysed baseline data (2015–2016) from the ELSI-Brazil cohort, a representative sample of non-institutionalised Brazilians aged ≥ 50 years. In total, 8807 individuals aged ≥ 50 years were included. Poisson regression with robust variance adjusted for confounders was used to verify the associations with hospitalisation. Multiple linear regression was used to analyse the associations with readmission and length of stay. Network analysis was conducted using 19 morbidities and the outcome variables. In 8807 participants, the prevalence of hospitalisation was 10.0% (95% CI 9.1, 11), mean readmissions was 1.55 ± 1.191, and mean length of stay was 6.43 ± 10.46 days. Hospitalisation was positively associated with male gender, not living with a partner, not having ingested alcoholic beverages in the last month, and multimorbidity. For hospital readmission, only multimorbidity ≥ 3 chronic conditions showed a statistically significant association. Regarding the length of stay, the risk was positive for males and negative for living in rural areas. Five disease groups connected to hospitalisation, readmission and length of stay were identified. To conclude, sociodemographic variables, such as gender, age group, and living in urban areas, and multimorbidity increased the risk of hospitalisation, mean number of readmissions, and mean length of stay. Through network analysis, we identified the groups of diseases that increased the risk of hospitalisation, readmissions, and length of stay.

## Introduction

Population ageing and its associated increased burden of chronic conditions are a challenge for health systems worldwide^1^. Hospitalisation is part of treatment actions for these chronic conditions when aggravated and its prevalence varies widely. For example, from 10.2% in Brazilians aged ≥ 50 years to 25.8% in older Portuguese adults aged 65 years or older^2,3^. Although often necessary, hospitalisation at old age has negative consequences such as decreased functionality, falls, poor mental health, risk of infections and worsening of other chronic conditions impacting on quality of life^4,5^.

The occurrence of multiple chronic conditions in the same individual is called multimorbidity^6^. Currently, multimorbidity is defined as the occurrence of ≥ 2 or ≥ 3 chronic conditions^7^. The occurrence of multimorbidity is higher in older adults, where its prevalence ranges from 55.0 to 98.0%^8^. A study in 17 European countries identified a wide variation in the prevalence of multimorbidity in adults aged ≥ 50 years. The lowest prevalence was found in Switzerland (20.0%) and the highest in the Czech Republic (39.5%)^9^. In Brazil, the prevalence of multimorbidity in individuals aged 50 years or older is 67.8% for ≥ 2 and 47.1% for ≥ 3 chronic conditions^10^.

Previous research has found that individuals with multimorbidity have an increased risk of poorer quality of life, higher frequency and costs of health services use and mortality^11–13^. Some studies have analysed the association between multimorbidity with hospitalisation, readmission, and length of stay in older adults, finding that multimorbidity may increase the risk of hospitalisation, readmissions, and length of stay^11^. In Switzerland, the prevalence of hospitalisation among older adults was 22.0% higher in those with multimorbidity, while length of stay doubled among them^14^. In another study conducted in 16 European countries, the occurrence of multimorbidity in individuals aged 50 years and older was associated with 1.49 times higher hospitalisation rates^15^.

Recent studies have observed that chronic conditions group together, forming patterns of multimorbidity^16^. To analyse these patterns, methods such as factor analysis, hierarchical clustering algorithm, unified clustering algorithm, multiple correspondence analysis, and network analysis have been used^17^. Factor analysis was used in most studies to identify groups of diseases associated with hospitalisation^18,19^. Network analysis has already been used in some studies on multimorbidity, however, the outcome of hospitalisation has not been used to elucidate the associations of multimorbidity with occurrence of hospitalisation^20,21^. Network analysis allows to verify simultaneous actions among the variables and thus capture the complexity of the data^22^. Previous studies have assessed hospitalisation and other health service utilisation^18,19^, already hospitalised individuals^23^, older adults aged ≥ 85 years^24^, and unplanned hospitalisations^25^. However, little is known about the patterns of multimorbidity associated with hospitalisation, readmission and hospital days in individuals aged 50 years and older.

Estimating the prevalence, associated factors, and multimorbidity patterns associated to hospitalisation, readmission, and length of stay in individuals aged ≥ 50 years will help understanding associated problems. This, in turn, may result in improvements in care provided, as well as the prevention of diseases and reduction of hospitalisations. Therefore, the objectives of this study were: (i) to analyse the prevalence of hospitalisation, mean occurrence of readmissions, and mean length of stay in Brazilian adults and older adults; (ii) to determine the factors associated with these three outcomes, especially the occurrence of multimorbidity with 2 and ≥ 3 chronic conditions; and (iii) to identify through network analysis the patterns of morbidities associated with hospitalisation, readmission, and length of stay in Brazilian adults and older adults.

## Methods

### Study population

Baseline data were drawn from the Brazilian Longitudinal Study of Ageing (ELSI-Brazil) between 2015 and 2016, involving community-dwelling individuals aged 50 years or older. The baseline of the study comprised 9412 participants living in 70 municipalities of the five great geographical Brazilian regions. The informed consent was obtained from all subjects. This study involved human beings, therefore, the ethical precepts of research with human beings based on the Declaration of Health (Declaration of Helsinki), and the resolutions of the National Health Council (196/96, 292/99, 340/2004, 346/2005, 347/2005, and 466/2012). ELSI-Brazil was approved by the Institution’s Ethics Committee (protocol n. 34649814.3.0000.5091). More details on the survey can be found on the ELSI-Brazil homepage^10,26^.

### Outcomes

The outcome variables were hospitalisation, readmission, and length of stay during the last 12 months. Hospitalisation was evaluated with the yes/no question, ‘In the past 12 months, have you been admitted to a hospital for 24 h or more?’ ‘Readmission’ indicates the number of times the individual was hospitalised in the past 12 months. ‘Days of hospitalisation’ refers to the number of days hospitalised during the last hospitalisation.

### Independent variables

The independent variables were: sex (female, male); age group (50–59 years, 60–74 years, and 75 or more); skin colour (brown, white, black/yellow/indigenous); living with a partner (no, yes); schooling years (illiterate, 1–4 years, 5–8 years, 9 years or more); asset index (quintiles), area of residence (urban, rural); household served by the Family Health Strategy (no, yes, does not know); alcohol consumption per month (never, less than once, once or more); health insurance (no, yes); and smoking status (does not smoke, current smoker, former smoker).

The asset index was calculated using Principal Component Analysis (PCA) with data from 12 variables related to household socioeconomic conditions such as number of assets and appliances, and number of employees in the household. The categories of the variable were created from the quintiles of the score generated by the analysis: 1st [0.83; 1.30]; 2nd [1.30; 1.38]; 3rd [1.38; 1.46]; 4th [1.46; 1.61]; 5th [1.61; 3.24]^27^. We set the fourth quintile was the reference category since it was the category with the lowest prevalence of hospitalisation. The Brazilian Family Health Strategy (FHS) programme was implemented to re-organise the primary health care of the country, according to the core principles of the Unified Health System (SUS) i.e. universality, accessibility, completeness of care, with a special focus on health promotion and disease prevention^28^.

Multimorbidity was assessed from a list of 19 morbidities and defined as: 2 morbidities and ≥ 3 morbidities^10^. The participants were asked: ‘Has a doctor ever told you that you have…?’ with the response options: no, yes, and don’t know/no answer for the 19 items on the list. In the network analyses, morbidities were investigated separately, with binary values (i.e. 0 = no, 1 = yes) for each variable.

### Statistical analysis

Of the 9412 individuals who participated in the baseline, 605 (6.4%) had missing data on at least one variable of interest. The Inverse Probability Weighting procedure was performed to assess the probability that each observation was a complete case^29^. After weighting, missing data were excluded from the database, resulting in a final sample of 8807 individuals. Analyses were performed in R software version 4.0.3^30^, considering the primary sampling unit (PSU) variables, stratification, and weights calculated for the sample, which allowed the calculation of proportions and adjusted 95% confidence intervals (95% CI). To measure the associations between the independent variables and the outcome of hospitalisation (yes/no), Poisson regression models with robust variance were estimated and adjusted for explanatory variables, and prevalence ratios (PR) and a 95% confidence interval (CI) were calculated. The associations between the independent variables and the readmission and length of stay outcomes were estimated through multiple linear regression models, adjusted for explanatory variables, and the β coefficients and 95% CIs were calculated.

#### Network analysis

The network analysis with the outcome hospitalisation and multimorbidity was estimated with the Ising model. The networks for readmission and length of stay were estimated with mixed graph models, which are appropriate for databases with different types of variables^31^. In all three networks, edges are interpreted as associations between two nodes, ‘controlling’ for the effect of all other nodes in the network. LASSO (Least Absolute Shrinkage and Selection Operator) was used as the regularisation method and EBIC (Extended Bayesian Information Criteria) as the model selection method, with hyperparameter γ set to 0.25. Bootstrapping analyses were performed to verify the accuracy and stability of the networks (Supplementary Material)^32^.

The detection of groups of diseases, or communities, was performed using the walktrap algorithm^33^. Disease groups or communities are sets of nodes that are highly connected among themselves but that do not present strong connections with the other nodes in a network.

Node predictability determines how well a node is predicted by all other nodes connecting to that node and can serve as a measure of the practical significance of edges. As a measure of predictability, the proportion of variance explained for quantitative variables and the proportion of correct classification for qualitative variables were used^34^. The size of each node represents the prevalence of each variable, and the thickness of the edges represent the connection between the nodes, such that the thicker the node, the more connected it is. Finally, the flow algorithm was used, enabling the visualisation of the network structure from a target node. The target node is positioned on the left of the network, and the other nodes are organised in layers according to the connections established with the target node. The variables related to hospitalisation were chosen as target nodes in all networks.

## Results

Of the 8807 Brazilian adults and older adults, 53.7% were women, 51.7% were aged 60 years or older, 20.7% had two chronic conditions, and 47.1% had multimorbidity of ≥ 3 conditions (Table 1). The prevalence of hospitalisation was 10.0% (9.1, 11.0), the mean readmission was 1.55 ± 1.191 and mean length of stay was 6.43 ± 10.46 days.

**Table 1:** Prevalence of hospitalisation and its association with sociodemographic, lifestyle and multimorbidity variables in older adults from the ELSI-Brazil cohort (2015–2016).

Independent variables	*n*	% (95% CI)	Hospitalisation (n = 887)	
			% (95% CI)	PR (95% CI)
**Gender**				
Female	4960	53.7 (50.6, 56.8)	9.5 (8.4, 10.6)	1.00
Male	3847	46.3 (43.2, 49.4)	10.4 (9.0, 11.9)	1.41 (1.14, 1.74)*
**Age group (years)**				
50–59	3778	48.3 (44.1, 52.5)	8.6 (7.4, 9.9)	1.00
60–74	3624	38.3 (35.6, 41.0)	10.5 (9.0, 12.0)	1.01 (0.85, 1.20)
≥ 75	1405	13.5 (11.7, 15.5)	13.3 (11.1, 15.5)	1.12 (0.9, 1.39)
**Skin color**^**1**^				
Brown	4025	43.2 (39.1, 47.3)	9.6 (8.2, 10.9)	1.00
White	3355	41.3 (35.9, 46.9)	10.2 (8.8, 11.6)	1.08 (0.9, 1.3)
Black/yellow/indigenous	1126	12.3 (10.4, 14.6)	11.3 (9.3, 13.4)	1.13 (0.9, 1.44)
**Living with a partner**				
Yes	5129	63.7 (60.7, 66.6)	9.3 (8.2, 10.5)	1.00
No	3678	36.3 (33.4, 39.3)	11 (9.8, 12.3)	1.21 (1.02, 1.43)*
**Schooling years**^**2**^				
≥ 9	2201	27.4 (24.9, 29.9)	9.5 (7.9, 11.0)	1.00
Illiterate	1391	12.8 (10.6, 15.5)	12.8 (10.8, 14.9)	1.12 (0.86, 1.46)
1–4	3397	37.8 (35.6, 40.1)	9.5 (7.9, 11.1)	0.88 (0.68, 1.13)
5–8	1771	21.6 (19.4, 23.9)	9.7 (8.0, 11.3)	1.01 (0.82, 1.24)
**Asset index (quintiles)**				
4th [1.46; 1,61]	1799	21.9 (19.3, 24.8)	9.2 (7.5, 10.9)	1.00
1st [0.83; 1.30]	1737	17 (13.4, 21.3)	9.4 (7.8, 11.0)	0.95 (0.74, 1.22)
2nd [1.30; 1.38]	1750	18.8 (15.8, 22.2)	11.1 (9.3, 12.9)	1.14 (0.88, 1.48)
3rd [1.38; 1.46]	1742	19.5 (17.6, 21.7)	10.4 (8.1, 12.7)	1.12 (0.89, 1.41)
5th [1.61; 3.24]	1779	22.8 (19.7, 26.1)	9.8 (8.3, 11.3)	1.09 (0.85, 1.41)
**Place of residence**				
Urban area	7445	85 (79.7, 89.1)	9.7 (8.9, 10.5)	1.00
Rural area	1362	15 (10.9, 20.3)	11.2 (7.9, 14.5)	1.23 (0.92, 1.65)
**Family health strategy**				
Do not know	514	5.2 (4.0, 6.6)	7 (4.1, 9.8)	1.00
No	2219	25.9 (21.8, 30.5)	10.2 (8.5, 11.8)	1.48 (0.97, 2.26)
Yes	6074	68.9 (63.6, 73.7)	10.1 (9.0, 11.2)	1.47 (0.96, 2.26)
**Health plan**				
No	6710	74.9 (72.0, 77.6)	9.6 (8.5, 10.7)	1.00
Yes	2097	25.1 (22.4, 28.0)	11.0 (9.5, 12.6)	1.18 (0.97, 1.45)
**Alcohol consumption (per month)**				
One or more times	1854	23.5 (20.9, 26.4)	6.6 (5.4, 7.9)	1.00
Never	6448	70.5 (67.8, 72.9)	11.2 (10.0, 12.3)	1.66 (1.3, 2.11)*
Less than once	505	6 (5.3, 6.9)	8.9 (5.6, 12.1)	1.36 (0.93, 1.98)
**Smoking status**				
Current smoker	1501	17 (15.6, 18.5)	8.5 (6.8, 10.2)	1.00
Do not smoke	3993	45.7 (43.8, 47.7)	8.8 (7.6, 9.9)	0.92 (0.74, 1.15)
Former smoker	3313	37.2 (35.3, 39.3)	12.1 (10.6, 13.5)	1.2 (0.96, 1.5)
**Multimorbidity**				
≤ 1	2719	32.2 (30.1, 34.4)	5.5 (4.3, 6.7)	1.00
2	1789	20.7 (19.6, 21.9)	8.8 (6.9, 10.7)	1.58 (1.16, 2.17)*
≥ 3	4299	47.1 (44.8, 49.4)	13.5 (12.1, 14.9)	2.39 (1.88, 3.04)*

*95% CI* 95% confidence interval, *PR* prevalence ratio, *n* unweighted number of choices. Relative frequencies (%) and weighted confidence intervals. ^1^*n* = 8506. ^2^*n* = 8760. *p < 0.05.

Hospitalisation was positively associated with being man, not living with a partner, not having drunk alcoholic beverages in the last month, and multimorbidity. Regarding age, the prevalence of hospitalisation was 8.6% in those aged 50 to 59 years, 10.5% in those between 60 and 74 years, and 13.3% in those aged 75 years or older (Table 1). For hospital readmission, the multimorbidity ≥ 3 chronic conditions was the only one showing an association. Regarding length of stay, the risk was positive for men and negative for living in rural area (Table 2).

**Table 2:** Average readmission and length of stay and their association with sociodemographic, lifestyle and multimorbidity variables in older adults from the ELSI-Brazil cohort (2015–2016).

Independent variables	*n*	% (95% CI)	Readmission		Length of stay (days)	
			M (*SD*)	β (95% CI)	M (*SD*)	β (95% CI)
**Gender**						
Female	497	51.5 (46.9, 56.0)	1.5 (1.1)	1.00	4.8 (9)	1.00
Male	390	48.5 (44.0, 53.1)	1.6 (1.3)	0.22 (− 0.12, 0.55)	8.1 (11.6)	3.63 (2.07, 5.19)*
**Age groups (years)**						
50–59	329	41.8 (34.7, 49.2)	1.5 (0.9)	1.00	6.4 (11.5)	1.00
60–74	364	40.2 (34.9, 45.7)	1.4 (0.8)	− 0.13 (− 0.3, 0.05)	6.3 (10.5)	− 0.5 (− 2.63, 1.63)
≥ 75	194	18 (14.6, 22.1)	1.5 (0.8)	− 0.19 (− 0.42, − 0.04)	6.8 (7.4)	0.01 (− 2.6, 2.62)
**Skin color**^**1**^						
Brown	387	41.4 (35.5 47.5)	1.5 (1.0)	1.00	5.8 (6.9)	1.00
White	339	42.2 (35.5, 49.2)	1.6 (1.1)	0.14 (− 0.07, 0.36)	7.2 (13.6)	1.35 (− 0.63, 3.32)
Black/yellow/indigenous	136	14 (10.5, 18.4)	1.7 (1.7)	0.24 (− 0.31, 0.78)	6.6 (8.6)	0.87 (− 1.05, 2.8)
**Living with a partner**						
Yes	485	59.8 (54.9, 64.4)	1.6 (1.2)	1.00	6.4 (10.4)	1.00
No	402	40.2 (35.6, 45.1)	1.5 (1.2)	0.05 (− 0.16, 0.27)	6.4 (10.6)	0.99 (− 0.47, 2.45)
**Schooling years**^**2**^						
≥ 9	199	26 (21.5, 31.0)	1.5 (1.2)	1.00	5.5 (7.0)	1.00
Illiterate	171	16,6 (13.1, 20.8)	1.7 (1.3)	0.2 (− 0.2, 0.61)	5.7 (7.3)	1.1 (− 1.39, 3.59)
1–4	338	36 (30.7, 41.7)	1.6 (1.3)	0.14 (− 0.19, 0.47)	6.9 (13.7)	2.07 (− 0.71, 4.85)
5–8	174	20.9 (17.1, 25.3)	1.3 (0.8)	− 0.18 (− 0.47, 0.1)	7.2 (9.6)	1.66 (− 0.19, 3.52)
**Asset index (quintiles)**						
4th [1.46; 1.61]	168	20.3 (16.1, 25.3)	1.5 (1.1)	1.00	6.6 (10.9)	1.00
1st [0.83; 1.30]	183	16 (12.0, 21.1)	1.5 (1.0)	− 0.04 (− 0.33, 0.24)	5.9 (7.1)	0.31 (− 1.68, 2.3)
2nd [1.30; 1.38]	188	20.9 (16.1, 26.7)	1.7 (1.5)	0.15 (− 0.29, 0.58)	6.2 (10.9)	0.15 (− 2.45, 2.76)
3rd [1.38; 1.46]	182	20.4 (16.5, 25.0)	1.5 (1.3)	0.01 (− 0.24, 0.26)	7.4 (14.1)	1.17 (− 1.6, 3.95)
5th [1.61; 3.24]	166	22.3 (18.0, 27.2)	1.5 (0.9)	0.01 (− 0.29, 0.32)	6.1 (7.3)	− 0.55 (− 3.17, 2.06)
**Place of residence**						
Urban area	748	83.1 (75.0, 89.0)	1.5 (1.1)	1.00	6.8 (11.2)	1.00
Rural area	139	16.9 (11.0, 25.0)	1.8 (1.6)	0.2 (− 0.25, 0.65)	4.7 (5.7)	− 2.68 (− 4.42, -0.94)*
**Family health strategy**						
Do not know	45	3.6 (2.2, 5.9)	1.4 (0.8)	1.00	7.6 (8.1)	1.00
No	219	26.4 (21.1, 32.6)	1.6 (1.4)	0.22 (− 0.21, 0.66)	5.6 (9.7)	− 1.53 (− 3.85, 0.79)
Yes	623	69.9 (63.5, 75.7)	1.5 (1.1)	0.15 (− 0.18, 0.49)	6.7 (10.9)	− 0.21 (− 2.48, 2.07)
**Health plan**						
No	653	72.2 (67.8, 76.2)	1.5 (1.1)	1.00	6.4 (9.3)	1.00
Yes	234	27.8 (23.8, 32.2)	1.6 (1.3)	0.06 (− 0.36, 0.47)	6.5 (13.0)	1.1 (− 1.38, 3.58)
**Alcohol consumption (per month)**						
One or more times	122	15.7 (12.6, 19.4)	1.3 (1.0)	1.00	7.1 (10.0)	1.00
Never	723	78.9 (74.7, 82.6)	1.6 (1.2)	0.23 (0.0, 0.45)	6.3 (9.5)	0.44 (− 1.81, 2.69)
Less than once	42	5.4 (3.7, 7.8)	1.5 (0.9)	0.18 (− 0.25, 0.6)	6.6 (20.6)	1.12 (− 3.89, 6.12)
**Smoking status**						
Current smoker	133	14.6 (11.7, 17.9)	1.4 (0.7)	1.00	6.3 (9.8)	1.00
Do not smoke	373	40.3 (36.3, 44.5)	1.7 (1.5)	0.31 (0.0, 0.62)	5.9 (10.3)	0.38 (− 2.11, 2.86)
Former smoker	381	45.1 (40.8, 49.5)	1.5 (1.0)	0.09 (− 0.07, 0.26)	6.9 (10.8)	0.53 (− 1.84, 2.9)
**Multimorbidity**						
≤ 1	162	17.8 (14.2, 22.2)	1.3 (1.1)	1.00	6.3 (12.0)	1.00
2	153	18.4 (14.8, 22.5)	1.3 (0.6)	0.01 (− 0.23, 0.24)	7.6 (12.4)	1.49 (− 1.49, 4.47)
≥ 3	572	63.8 (58.9, 68.5)	1.7 (1.3)	0.34 (0.15, 0.53)*	6.1 (9.3)	0.39 (− 1.65, 2.43)

*95% CI* 95% confidence interval, *n* unweighted number of observations. Relative frequencies (%) and weighted confidence intervals. *M* mean, *SD* Standard deviation. ^1^*n* = 862. ^2^*n* = 882. *p < 0.05.

**Figure 1:**
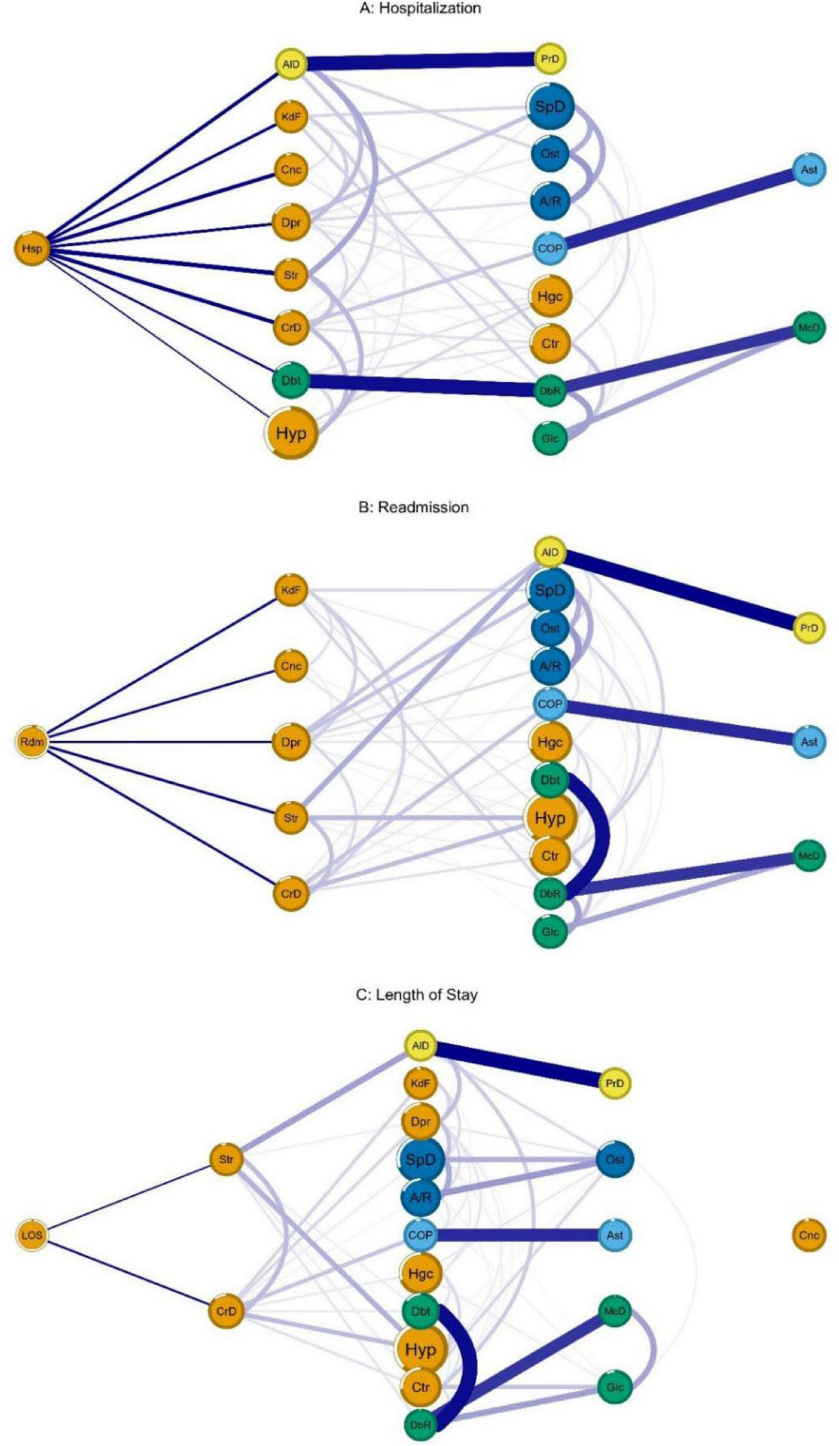
Flow networks of morbidities and hospitalisation variables. The Brazilian Longitudinal Study of Ageing (ELSI-Brazil), 2015—2016. Notes. Disease groups: *Cardiometabolic diseases, cancer and others* (orange colour): Ctr (Cataract), Hyp (Hypertension), Hgc (High Cholesterol), CrD (Heart Disease), Str (Stroke), Dpr (Depression), Cnc (Cancer), KdF (Kidney Failure), Hsp/Rdm/DoH (hospitalisation/Readmission/Length of stay); *Respiratory diseases* (blue colour): Ast (Asthma), COP (Chronic obstructive pulmonary disease); *Diabetes and its complications* (green colour): Glc (Glaucoma), DbR (Diabetic Retinopathy), McD (Macular Degeneration), Dbt (Diabetes); *Neurodegenerative diseases* (yellow colour): PrD (Parkinson Disease), AID (Alzheimer Disease); *Musculoskeletal disorders* (dark blue colour): A/R (Arthritis/Rheumatism), Ost (Osteoporosis), SpD (Spine Problem).

The network analysis indicated that the most prevalent morbidities were hypertension (52.0%), back problems (40.3%), high cholesterol (30.0%), cataract (24.4%), and arthritis/rheumatism (20.7%) (Table 3). Regarding the analysis of groups of diseases, the following were identified: cardiometabolic diseases, cancer, and others (orange colour: stroke, heart disease, hypertension, high cholesterol, renal failure); respiratory diseases (light blue colour: asthma and COPD); diabetes and its complications (green colour: glaucoma, diabetic retinopathy, macular degeneration, and diabetes); neurodegenerative diseases (yellow colour: Alzheimer’s disease and Parkinson’s disease); and musculoskeletal diseases (dark blue colour: arthritis/rheumatism, osteoporosis, and back problem). The group of cardiometabolic diseases, cancer, and others also included the variables of hospitalisation, readmission, and length of stay, suggesting a greater impact on these variables (Fig. 1).

**Table 3:**
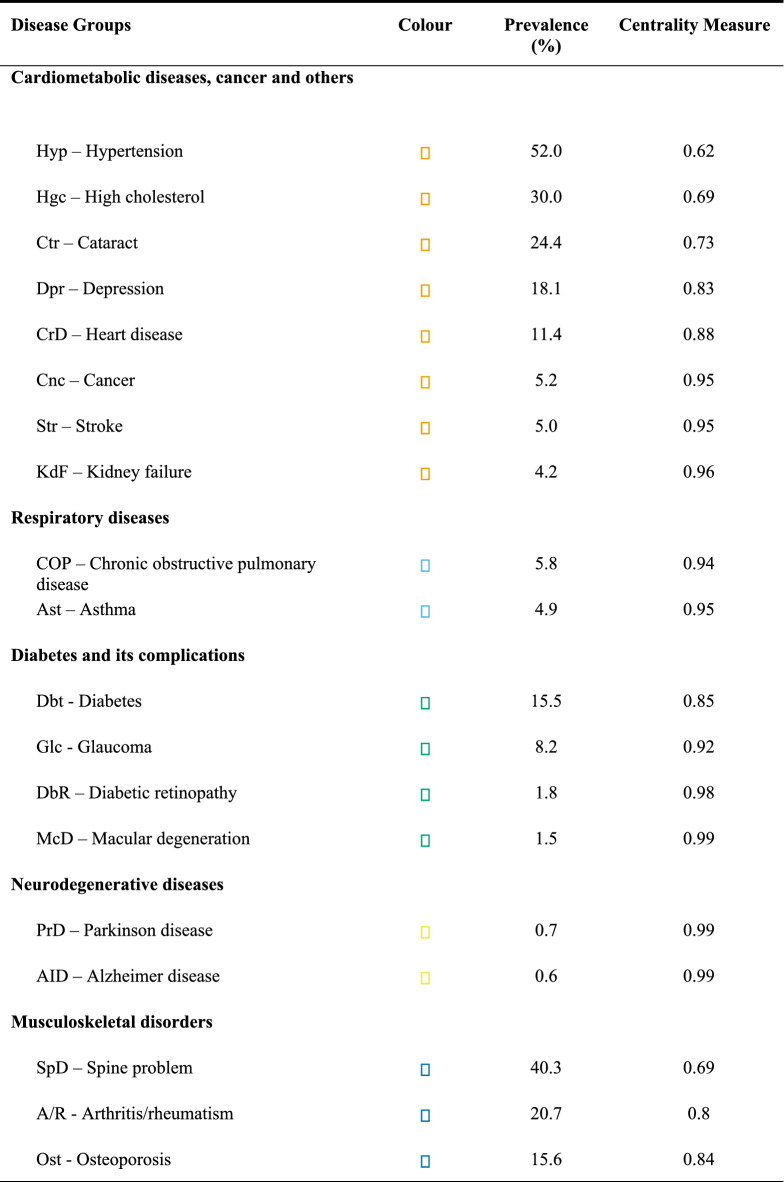
Prevalence and centrality measure of hospitalisation network nodes, readmission and length of stay. The Brazilian Longitudinal Study of Ageing (ELSI-Brazil), 2015–2016.

In the association networks (Fig. 1), the nodes further to the left present direct connectivity with hospitalisation, readmission, and length of stay. The morbidities directly connected with hospitalisation in network A (Fig. 1) were as follows: Alzheimer’s disease, heart failure, cancer, depression, stroke, heart disease, diabetes, and hypertension. These morbidities act as mediators between those located further to the right of the network with hospitalisation. For example, Alzheimer’s disease mediated between Parkinson’s disease and hospitalisation in the same way that diabetes mediated the association between diabetic retinopathy and hospitalisation.

In Network B, after the analysis of hospital readmission (Fig. 1), we observed a change in the pattern of morbidities associated with readmission in relation to the pattern in the hospitalisation network because the directly connected morbidities were renal insufficiency, cancer, depression, stroke, and heart disease. In this network, stroke acted as a mediator between Alzheimer’s disease and hypertension in relation to the occurrence of readmission.

In the C network, after the analysis of length of stay, we observed that the number of morbidities directly connected to this outcome, length of stay, was related to a smaller number of morbidities i.e. stroke and heart disease only. Based on the thickness of the edges, we observed that stroke was strongly associated with Alzheimer’s disease, hypertension, and heart disease (Fig. 1).

## Discussion

This is the first study in Latin America to analyse the associations with hospitalisation, readmissions, and length of stay in relation to patterns of morbidities using a network analysis approach. This type of analysis allowed us to explore and understand the complexity of multimorbidity and its impact on hospitalisation. The highest risk of hospitalisation was for men, not living with a partner, not having drunk alcoholic beverages in the last month, and multimorbidity (2 and ≥ 3 morbidities). Readmissions were associated only with multimorbidity ≥ 3 and the outcome length of stay was positively associated with being male and negatively associated with living in rural areas. Multimorbidity was associated with both hospitalisation and readmission. The network analysis helped identifying five groups of diseases with the ‘cardiometabolic, cancer, and others’ group being associated with hospitalisation, readmission, and length of stay.

The prevalence of hospitalisation found (10.0%) was similar to the one from a Chinese study (10.5%)^35^. The mean length of stay found (6.43) was lower than the one found in the population aged 65 years and older in a Swedish study, which was 8.10^36^. Regarding readmission (mean 1.55), in the population aged ≥ 65 years from 29 primary health care centres in Sweden, the mean was 7.7^37^. In our study, the prevalence of hospitalisation was not as high as that found by the Brazilian Hospital Information System in 2019 (37.4%)^38^ in the same age group.

The association between hospitalisation and male gender observed in our study was similar to that found by other studies conducted in older American adults^39^. However, in China this association was not observed in individuals aged ≥ 45 years^40^. This result can be interpreted as women use health services more frequently for consultations and preventive examinations, while men tend to seek health care only when they present more severe symptoms that can already lead to hospitalisation^41^. This highlights the importance of public policies aimed at preventive measures for men’s health. Living alone was associated with hospitalisation in our study. This result was similar to a systematic review that included 35 studies conducted in older adults with multiple chronic conditions^11^. Having a support network is important for the maintenance and care of health. People who live alone and have multimorbidity may have difficulty in managing their own health, thus increasing their risk of hospitalisation.

Living in rural areas was a protective factor for length of stay. However, since we did not find previous studies that evaluated the association between area of residence and length of stay, this is a relevant finding. We interpreted this result based on two hypotheses, the first being that individuals who live in rural areas are healthier, and the second that when they are hospitalised, they present less severity of illness, leading to faster discharge than those living in urban areas. Further studies are needed.

Not having consumed alcoholic beverages in the last month was associated with hospitalisation in our study. A previous study conducted in China also found that older adults who did not consume alcoholic beverages had higher rates of hospitalisation. Our finding can be explained by behavioural changes that tend to occur among older adults after they present health problems due to drinking^40^. Thus, this association may be an example of reverse causality since our study had a cross-sectional design. In addition, the Goodman–Kruskal Gamma test was performed to better understand this association between non-intake of alcoholic beverages and hospitalisation. Among those who had never drunk alcoholic, there was a higher probability of having ≥ 3 multimorbidity, while in those who had consumed alcohol once or more per month, there was an association with ≤ 1 morbidity, confirming our hypothesis of reverse causality.

Multimorbidity, either 2 or ≥ 3, was associated with hospitalisation, readmission, and length of stay. This result is similar to previous studies that found an increase in the number of chronic conditions associated with hospitalisation^14,15^. These results further highlight the importance of effective actions of the Family Health Strategy programme, such as prevention, health promotion, and treatment. Such actions, especially among younger older adults who are more likely to be in the early stages of chronic conditions, reduce hospitalisations and even premature deaths^42^.

The most prevalent chronic conditions in our study were hypertension, back problems, and high cholesterol. In the United States, a high prevalence of hypertension (56.2%) and high cholesterol (42.8%) were also found among Medicare beneficiaries^43^. Back problems are an infrequent condition in the list of diseases assessed in most studies. Moreover, the prevalence of this condition in our study (40%) was higher than that found in the general population of Lithuania which was 4.21%^44^. These results are important to guide primary health care actions to focus on these morbidities which in turn can help improve clinical protocols and promote health.

Despite the differences found in the networks of connections of morbidities with hospitalisation, readmission, and length of stay, we observed that the group of cardiometabolic diseases, cancer, and other remained associated with the three outcomes analysed. These results are in line with an American cohort that showed an association between hospitalisation and cardio-stroke-cancer^45^. In Spain, the clusters of cardiorespiratory/mental/arthritis and metabolic/stroke were associated with hospitalisation^18^. While a study conducted in Italians aged 65 years and older, the cardiac disease and metabolic/ischemic patterns were associated with a higher risk of readmission, and the cardiac disease pattern showed higher mean length of stay^19^. The fact that most morbidities of this group (cardiometabolic, cancer and others) share common risk factors such as lifestyle reinforces the importance of health promotion actions focused on primary health care aimed at this group of diseases in order to reduce and prevent hospitalisations.

In the network analysis, we observed that depression and cataract belonged to the group of cardiometabolic diseases and were associated with increased hospitalisation, readmission, and length of stay. In other studies, depression was associated with other chronic conditions such as cardiometabolic conditions^46,47^. A study in older adults with a mean age of 67 years identified depression as increasing hospital readmission rates among those with cardiovascular disease^47^. It is important to identify and treat depression, as it can be both a cause and consequence of hospitalisation. However, due to the design of this study, the causality between these variables, depression, and hospitalisation could not be determined. Regarding cataract, as in our study, a Spanish cohort identified a high prevalence of this condition in the ‘cardiovascular/mental/arthritis’ and ‘metabolic/CVA’ disease clusters^18^.

In the hospitalisation network, Alzheimer’s disease stood out as a mediator between Parkinson’s disease and hospitalisation. People with Alzheimer’s disease have a high risk of being hospitalised^48^. Another Brazilian study found that Alzheimer’s disease increases the likelihood of hospitalisation, as well as increasing the days hospitalised and the chance of being diagnosed with another chronic condition such as depression or Parkinson’s disease^49^. People with Alzheimer’s have worse health outcomes and are at greater risk of hospitalisation, readmission, increased hospitalisation days and mortality. Therefore, improving the screening of these individuals and preventive measures aimed at the Brazilian National Primary Health Care strategy are necessary to avoid common complications that cause hospitalisation^50^.

In the networks of hospitalisation and readmission outcomes, the directly connected morbidities were practically the same, while in the network of length of stay, only two morbidities were directly connected i.e. heart disease and stroke. An Italian cohort study of patients with complex healthcare needs aged 65 years and older identified that those with heart failure, ischemic, or cardiorespiratory disease groups were significantly more likely to spend more days in hospital^19^. In our study, cancer was directly connected to hospitalisation and readmission, but in the hospitalisation days network, this morbidity was isolated from the rest of the network. This result was also found in an Italian study in which cancer was not associated with length of stay^19^. Considering that the longer the length of stay, the greater the negative consequences^51^, the identification of chronic conditions directly associated with this outcome allows professionals and health services to improve the care and treatment of these people.

ELSI-Brazil, with a representative sample of community-dwelling individuals aged 50 years and older, allows us to understand issues that pervade the ageing process and its implications for health. Consequently, our findings allow generalisation, adding internal and external validity to the theme investigated herein. The network analysis enabled us to visualise the relationships between hospitalisation, readmission, and length of stay, and the occurrence of multimorbidity.

As a limitation, we mention the reverse causality that is characteristic of the lack of temporality of cross-sectional studies, which implies difficulty in understanding certain associations. However, the second wave ELSI-Brazil data is going to be publicly available soon, allowing us to explore this topic longitudinally. In addition, the list of diseases used in this study includes chronic conditions only. Some acute conditions were not included, such as respiratory and urinary tract disorders, which may cause frequent hospitalisations in this age group^52^. Alcohol consumption was measured only in relation to the month before the interview and, therefore, there may have been an underestimation of the true proportion of alcohol consumption among participants, and, consequently, its association with the outcomes for hospitalisation. Another limitation relates to the chronic diseases and primary outcomes (hospitalisation, readmission, and length of stay) data being self-reported, since this could be a potential source of bias (i.e. recall bias). We did not have information on the duration of all chronic diseases included in this study, and whether these diseases were controlled and monitored by health professionals. Finally, there was no information on the number of days of inappropriate hospital stay at a higher cost than an adequate hospital stay with fewer days.

Future research should apply multimorbidity network analysis to hospitalisation and other outcomes as a way of broadening knowledge in this area. Cohort studies will be important, as well as nationally representative studies like ours to expand the knowledge of hospitalisation and multimorbidity. Our results may contribute to the improvement of public health strategies. Actions focused on primary health care to better prevent and manage multimorbidity, especially conditions related to cardiometabolic diseases and cancer, may result in less hospitalisation.

## Conclusions

Hospitalisation was associated with male gender, not living with a partner, not having consumed alcohol in the last month and multimorbidity (2 and ≥ 3 morbidities). Readmission was associated only ≥ 3 morbidities. Mean length of stay was higher in men and lower in those living in rural areas. A network analysis was applied, and five groups of diseases were identified: cardiometabolic, cancer, and other; respiratory diseases; diabetes and its complications; neurodegenerative diseases; and musculoskeletal diseases. Morbidities tended to group together, increasing the risk of hospitalisation, readmission, and length of stay. The ‘cardiometabolic diseases, cancer, and others’ group was strongly associated with hospitalisation, readmission, and length of stay.

## Supplementary Information

Supplementary Figure S1.

## Data Availability

Access to data on the website: http://elsi.cpqrr.fiocruz.br/.
