# Supplementary material for: Multimorbidity patterns and hospitalisation occurrence in adults and older adults aged 50 years or over
Source: Sci Rep. 2022 Jul 8;12:11643. doi: 10.1038/s41598-022-15723-4 (PMC9270321; doi:10.1038/s41598-022-15723-4)
Supplement: Supplementary file 1 — Supplementary Figure S1. [file 41598_2022_15723_MOESM1_ESM.pdf]

Figure S1. Measures of morbidity centrality and hospitalisation variables. The Brazilian Longitudinal Study of Ageing (ELSI-Brazil), 2015 - 2016.

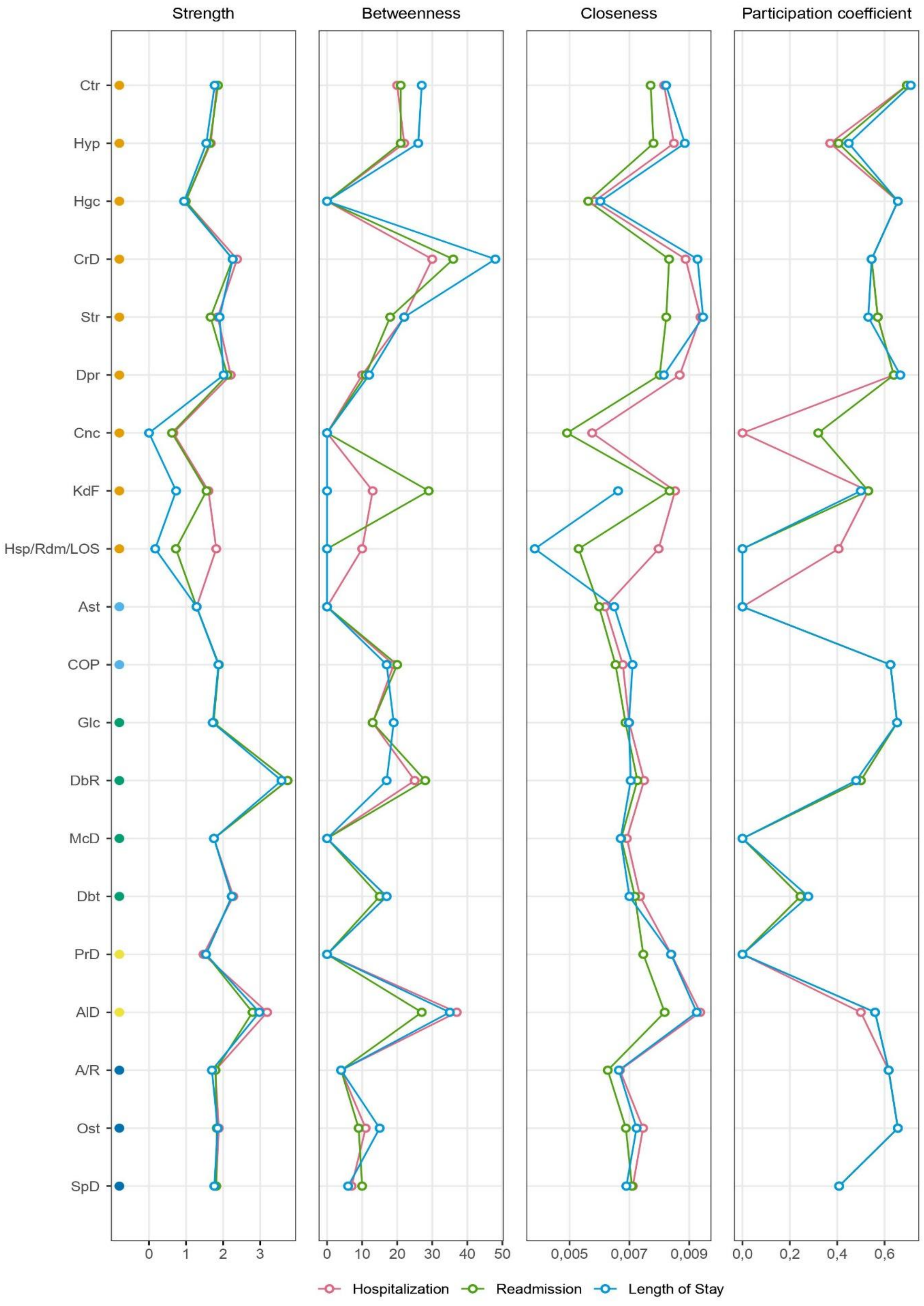

Notes. Disease groups: *Cardiometabolic diseases, cancer and others* (orange colour): Ctr (Cataract), Hyp (Hypertension), Hgc (High Cholesterol), CrD (Heart Disease), Str (Stroke), Dpr (Depression), Cnc (Cancer), KdF (Kidney Failure), Hsp / Rdm / DoH (Hospitalization / Readmission / Length of stay); *Respiratory diseases* (blue colour): Ast (Asthma), COP (Chronic obstructive pulmonary disease); *Diabetes and its complications* (green colour): Glc (Glaucoma), DbR (Diabetic Retinopathy), McD (Macular Degeneration), Dbt (Diabetes); *Neurodegenerative diseases* (yellow colour): PrD (Parkinson Disease), AID (Alzheimer Disease); *Musculoskeletal disorders* (dark blue colour): A/R (Arthritis / Rheumatism), Ost (Osteoporosis), SpD (Spine Problem).
